# Admission creatinine and outcomes in very elderly critically ill patients: a retrospective cohort study

**DOI:** 10.1186/s12877-026-07793-0

**Published:** 2026-06-10

**Authors:** Alexandre Klopp, Silvia de Rosa, Elisa Alba Schmidt, Markus Haar, Anna Carola Hertrich, Christina Thompson, Rikus Daniels, Pauline Theile, Markus Goedel, Stefan Kluge, Tobias B. Huber, Jakob Müller, Kevin Roedl, Christian Schmidt-Lauber

**Affiliations:** 1 III. Department of Medicine, University Medical Center Hamburg-Eppendorf, Hamburg, Germany; 2 Hamburg Center for Kidney Health (HCKH), University Medical Center Hamburg-Eppendorf, Hamburg, Germany; 3 Centre for Medical Sciences - CISMed, University of Trento, Trento, Italy; 4 Anesthesia and Intensive Care, Santa Chiara Regional Hospital, APSS Trento, Italy; 5 Department of Intensive Care Medicine, University Medical Center Hamburg-Eppendorf, Hamburg, Germany; 6 Department of Geriatrics, Albertinen Hospital, Hamburg, Germany; 7 Department of Anesthesia, Tabea Hospital, Hamburg, Germany

**Keywords:** Intensive care unit, Very elderly patients, Kidney function, Acute kidney injury, Creatinine, Nonagenarians

## Abstract

**Background:**

Serum creatinine at ICU admission is a central component of prognostic assessment in critically ill patients and is widely used as a marker of kidney function in clinical practice, as well as being incorporated into established prediction models. However, in very old patients, age-related muscle loss, frailty, and altered creatinine kinetics may limit its validity. With ICU admissions among nonagenarians (≥ 90 years) steadily increasing, it remains unclear whether admission creatinine retains its prognostic relevance in this population. We therefore evaluated the prognostic value of admission creatinine for short- and long-term outcomes in critically ill nonagenarians and assessed its role within the SOFA score.

**Methods:**

This retrospective cohort study included all patients aged ≥ 90 years admitted to the ICUs of the University Medical Center Hamburg-Eppendorf between 2008–2019. Admission serum creatinine was analyzed as the primary exposure variable. Primary outcomes were in-hospital, 90-day, and 1-year mortality. Associations between admission creatinine and outcomes were analyzed using logistic and Cox regression models adjusted for relevant confounders. To evaluate the prognostic contribution of kidney function to established risk scores, we compared the predictive performance of the full SOFA score with a modified SOFA score excluding the kidney (creatinine) component.

**Results:**

Among 952 patients (median age 92.2 years) in-hospital, 90-day, and 1-year mortality rates were 28.5%, 42.2%, and 57.1%, respectively. Admission creatinine was not independently associated with in-hospital mortality (adjusted hazard ratio [HR] 1.07, 95% CI 0.92–1.26). In contrast, higher admission creatinine independently predicted 90-day mortality (adjusted HR 1.16, 95% CI 1.01–1.34) and 1-year mortality (adjusted HR 1.20, 95% CI 1.06–1.36). The Full and non-kidney SOFA scores performed similarly (AUC 0.79 each).

**Conclusions:**

In critically ill patients aged ≥ 90 years, admission creatinine does not independently predict short-term outcomes but remains a robust predictor of mid- and long-term mortality. The unchanged performance of the SOFA score after removal of its kidney component underscores the limited relevance of creatinine for short-term risk stratification in this age group and supports age-adapted interpretation of kidney biomarkers and prognostic models in very old ICU patients.

**Supplementary Information:**

The online version contains supplementary material available at 10.1186/s12877-026-07793-0.

## Introduction

The global population is aging rapidly, and the number of octogenarians (80–89 years) and particularly nonagenarians (90–99 years) admitted to intensive care units (ICUs) is steadily increasing [1–4]. These patients often have complex medical histories, reduced physiological reserve, and atypical clinical manifestations, all of which complicate decision-making and accurate outcome prediction [5–7]. Moreover, the burden of chronic comorbidities, polypharmacy, and frailty often interacts with the acute illness, further complicating clinical trajectories [8]. Mortality rates remain high, and survivors frequently experience prolonged recovery, functional decline, and reduced quality of life [9]. In this context, reliable prognostic markers are essential to guide treatment strategies and facilitate informed discussions with patients and their families [10].

Among critically ill adults, serum creatinine, is a key prognostic biomarker and a widely used marker of kidney function, as well as an integral component of multiple prediction models, including the Sequential Organ Failure Assessment (SOFA) or the Acute Physiology And Chronic Health Evaluation (APACHE) II score, which are widely used to estimate in-hospital mortality [11, 12]. Elevated creatinine at ICU admission has consistently been associated with adverse short-term outcomes in mixed-age cohorts, reflecting its role as a marker of both kidney function and overall illness severity [13–16]. However, older adults exhibit substantial physiological changes, including a progressive decline in kidney function and muscle mass, both of which affect creatinine metabolism and kinetics, thereby limiting its role as a marker of kidney function in this population [17, 18]. Despite these limitations, creatinine indicators remain widely used as kidney function marker in very old patients. If creatinine retains its role as a key prognostic marker in critically ill nonagenarians remains insufficiently studied. This study aims to evaluate the prognostic value of serum creatinine at ICU admission—including its relevance within the SOFA score—for short- and long-term outcomes in critically ill patients aged ≥ 90 years. In addition, we sought to compare admission and pre-admission creatinine, which better reflects baseline kidney function and is preferred by current guidelines for the diagnosis of acute kidney injury [19], in terms of their association with outcomes and the definition of AKI in this vulnerable population. Clarifying the prognostic value of creatinine in very old ICU patients may have important clinical implications and may help inform future research directions. If creatinine remains strongly associated with outcomes, its continued use in risk stratification and established prognostic scores would be justified in this age group. Conversely, limited predictive value would suggest that current creatinine-based assessments may misclassify illness severity and underscore the need for alternative biomarkers or age-adapted prognostic approaches.

## Methods

### Study design and population

This retrospective cohort study included all individuals aged 90 years or older at the time of admission to the Department of Intensive Care Medicine at the University Medical Center Hamburg-Eppendorf, Germany, between January 1, 2008, and April 19, 2019. The department operates 12 intensive care units, encompassing a total capacity of 140 beds, and provides care for critically ill patients from both surgical and medical specialties. Patients without a creatinine measurement at ICU admission (admission creatinine) were excluded. In patients with multiple hospitalizations, the first admission was defined as the index hospitalization. Follow-up continued until hospital discharge, with survival monitored for up to one year following ICU admission. Ethical approval was granted by the local ethics committee, which waived informed consent due to the use of de-identified data (Approval No. 2022–300219-WF).

### Data acquisition

Clinical data were extracted from the hospital’s electronic patient data management system (PDMS, Integrated Care Manager (ICM), Version 9.1, Draeger Medical, Lübeck, Germany). Collected variables included demographics (age, sex), comorbidities, admission category (medical, elective surgical, emergency surgical), vital signs, interventions, laboratory values, pharmacological treatments, length of ICU and hospital stay, as well as discharge information. Laboratory testing was performed daily, and blood gas analyses were conducted at least three times per day using Atellica Solution platforms (Siemens Healthineers) and Radiometer ABL-90 analyzers (Krefeld, Germany). Creatinine was measured in heparin plasma or serum samples using a photometric assay (Atellica Solution CH930, Siemens Healthineers). 

### Definitions of predictors and outcomes

Admission creatinine was defined as the first serum creatinine measurement obtained within 24 h after ICU admission. Pre-admission creatinine was assessed through a joint clinical evaluation by an intensivist and a nephrologist, based on available pre-hospital medical records, and defined in accordance with KDIGO recommendations as the most recent creatinine value from the 7 days prior to ICU admission or, if no value was available in that timeframe, the most recent measurement within the previous 365 days [19].

The primary outcomes were all-cause in-hospital, 90-day and 1-year mortality during the follow-up period. Secondary outcomes included length of ICU and hospital stay, as well as a composite outcome of short-term adverse kidney events, defined as a de novo estimated glomerular filtration rate (eGFR) < 30 mL/min or kidney replacement therapy (KRT) dependency at discharge. eGFR was estimated using the CKD-EPI 2009 equation. Tertiary analyses assessed the predictive ability of the SOFA score with and without the inclusion of admission creatinine on in-hospital mortality. Here, the full SOFA score refers to the original score including all six organ system components, whereas the non-kidney SOFA score excludes the kidney/creatinine component. Lastly, we compared the association of admission and pre-admission creatinine on the primary outcomes, as well as the incidence, severity, and mortality of acute kidney injury (AKI) defined using the highest serum creatinine during ICU stay and admission or pre-admission creatinine as the reference for baseline kidney function. Analyses comparing admission and pre-admission creatinine were restricted to individuals with availability of both values (n = 856). AKI was defined according to the 2012 Kidney Disease: Improving Global Outcomes (KDIGO) criteria as an increase in serum creatinine ≥ 0.3 mg/dL within 48 h or a ≥ 1.5-fold rise within seven days as compared to the baseline kidney function. AKI severity was categorized as mild (1.5–1.9-fold increase), moderate (2–2.9-fold increase), or severe (≥ threefold increase, peak creatinine > 4 mg/dL, or initiation of KRT) [19].

### Statistical methods

Baseline characteristics reflect the status at ICU admission. Continuous variables are summarized as medians with interquartile ranges (IQR), and categorical variables as counts and percentages. Disease course variables included procedures and therapies administered during ICU care. Kaplan–Meier curves were used to illustrate survival according to admission creatinine, categorized into tertiles based on its distribution in the study population. Median survival times with 95% confidence intervals (CIs) were estimated for each tertile. Cox proportional hazards models were applied to evaluate in-hospital, 90-day and 1-year mortality, allowing incorporation of censoring and variable follow-up duration. Results are presented as hazard ratios (HRs) with 95% CIs. Linear regression models, reported as β-coefficients with 95% CIs, were used to assess hospital and ICU length of stay. All regression models used admission creatinine as a continuous linear variable. To ensure linearity, we additionally fitted Cox models using restricted cubic splines (three degrees of freedom). Model fit was compared to the corresponding linear specification using a likelihood-ratio test (LRT), with results presented in the Supplementary Material.

The prognostic contribution of the kidney component (admission creatinine) within the SOFA score was examined by comparing the full SOFA score (all six organ system components) with a modified non-kidney SOFA score (excluding the admission creatinine component). Both scores were analyzed for their association with in-hospital mortality using logistic regression, and for discriminative performance using receiver operating characteristic (ROC) curves with calculation of the area under the curve (AUC) and 95% CIs. In a subgroup of patients with both admission and pre-admission creatinine available (*n* = 856), we compared the associations of admission and pre-admission creatinine with mortality outcomes. Cox regression (for in-hospital and 1-year mortality) were performed in this subcohort.

Variables with non-normal distributions were log- or square root-transformed prior to regression analysis. All regression models were adjusted for potential confounders, selected according to a predefined protocol [20], including established ICU mortality risk factors: age, sex, body mass index (BMI), Charlson Comorbidity Index (CCI), Glasgow Coma Scale (GCS), bilirubin at admission, use of vasopressors, mechanical ventilation during ICU stay, admission type, lactate levels, and mean arterial pressure (MAP) at admission. For sensitivity analyses renal replacement therapy (RRT) or AKI were additionally adjusted for as potential confounders in our regression models. Baseline characteristics were compared between patients included in the analysis and those excluded due to missing admission creatinine measurements to assess potential differences between the groups.

Missing data were handled via random forest imputation using the missForest package (version 1.4) in R. All statistical analyses were performed using R (version 4.3.0).

## Results

### Study population and baseline characteristics

Of 92,958 ICU admissions during the study period, 1,125 admissions aged ≥ 90 years. After excluding individuals without available admission creatinine measurements and restricting the analysis to the first hospitalization per patient, 952 patients comprised the final study cohort (Supplementary Figure S1). Missing data were below 10% for most variables, with the exceptions of BMI (29%), bilirubin (25%) and temperature (18%) at admission and pre-admission creatinine (10%) (Supplementary Table S1). The median age of the study population was 92.2 years (IQR: 3.1), and 66.7% of the patients were female. The most common reason for ICU admission was elective surgery (37.8%), followed by medical (31.8%) and emergency surgical admissions (30.4%). The patients had a median creatinine value at admission of 1.1 mg/dL (IQR: 0.8 mg/dL). Additional patient characteristics at ICU admission are summarized in Table 1. During the ICU stay, 43.4% required vasopressor support, 34.5% underwent mechanical ventilation, 3.2% required KRT, and 3.4% received cardiopulmonary resuscitation (CPR).

**Table 1:** Patient characteristics at ICU admission

Characteristics	Overall (*n* = 952)
*Demographics*	
Age in years – median (IQR)	92.2 (3.1)
Female sex – n (%)	635 (66.7)
BMI in kg/m^2^ – median (IQR)	23.4 (3.9)
*Comorbidities*	
Charlson comorbidity index – median (IQR)	1 (2)
Hypertension – n (%)	674 (70.8)
eGFR < 60 mL/min/1.73 m^2^ – n (%)	668 (70.2)
Atrial fibrillation – n (%)	373 (39.2)
Cerebrovascular disease – n (%)	145 (15.2)
Diabetes – n (%)	129 (13.6)
Peripheral vascular disease – n (%)	84 (8.8)
COPD – n (%)	72 (7.6)
*Primary admission*	
Medical – n (%)	303 (31.8)
Elective surgery – n (%)	360 (37.8)
Emergency surgery – n (%)	289 (30.4)
*General presentation at admission*	
GCS – median (IQR)	15.0 (3)
Temperature in degrees Celsius – median (IQR)	36.2 (1)
MAP in mmHg – median (IQR)	85.0 (30.2)
Heart rate in bpm – median (IQR)	80.0 (37)
*Laboratory results at admission*	
Hemoglobin in g/dL – median (IQR)	10.3 (2.3)
Platelet count in 10^3^/microliter – median (IQR)	210 (116)
Sodium in mmol/L – median (IQR)	140 (5)
Creatinine in mg/dL – median (IQR)	1.1 (0.8)
Bilirubin in mg/dL – median (IQR)	0.7 (0.6)
Lactate in mmol/L– median (IQR)	1.1 (0.9)

*IQR* interquartile range, *BMI* body mass index, *COPD* chronic obstructive pulmonary disease, *GCS* Glasgow Coma Scale, *MAP* mean arterial pressure, *bpm* beats per minute

Although slight differences were observed in admission characteristics and lactate levels, included and excluded patients were otherwise largely comparable, suggesting a low likelihood of relevant selection bias (Supplementary Table S2).

### Association of admission creatinine with mortality

**Fig. 1:**
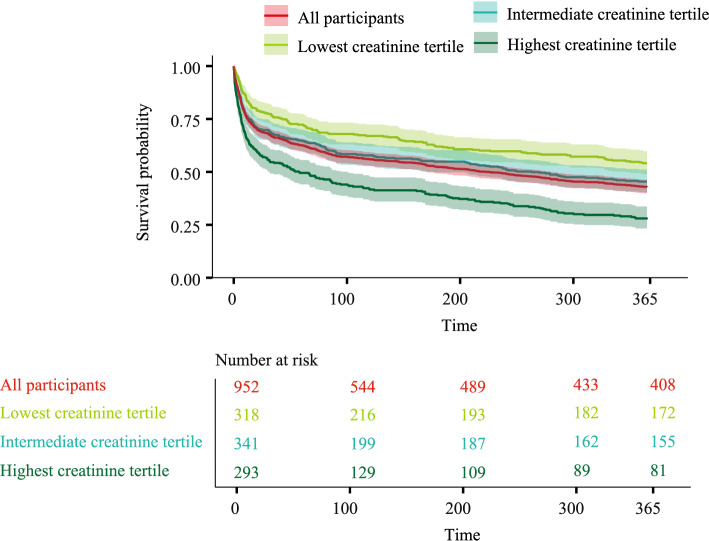
Survival probability according to admission creatinine-tertiles. Kaplan Meier curves showing survival probability until one year after ICU admission for all patients (red) and by admission creatinine tertiles (Low ≤ 0.89 mg/dL = light green, Medium > 0.89–1.40 mg/dL = light blue, High > 1.40 mg/dL = dark green). Shaded areas represent 95% confidence intervals (CI)

Of the 952 patients included in the analysis, 271 (28.5%) died during hospital stay, 402 (42.2%) died within 90 days, and 544 (57.1%) died within one year after ICU admission. Survival curve analyses revealed a high mortality during the first 50 to 100 days as well as a graded decrease in 1-year survival across admission creatinine tertiles (low ≤ 0.89 mg/dL, medium > 0.89–1.40 mg/dL, high > 1.40 mg/dL) with survival probabilities of 54.1% in the lowest, 45.5% in the middle, and 27.6% in the highest tertile (Fig. 1).

Associations of admission creatinine with outcomes were further investigated in regression models analyzing creatinine as a continuous linear variable. Nonlinear modeling using restricted cubic splines showed no evidence of deviation from linearity between log-transformed admission creatinine and 1-year mortality (likelihood-ratio test comparing spline and linear models: Δχ^2^ = 0.09, *p* = 0.95) and all spline terms were non-significant (*p* > 0.05) (Supplementary Figure S2 and Supplementary Table S3).

**Fig. 2:**
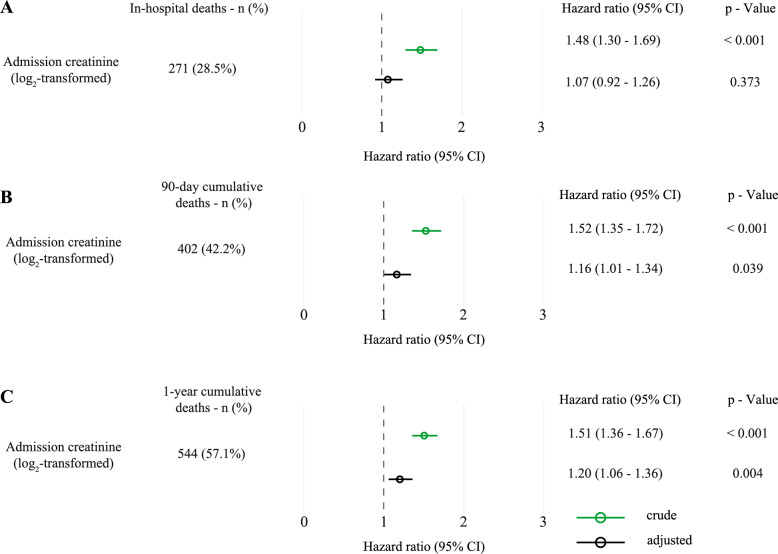
Association of admission creatinine with mortality. Forest plots showing the associations between admission creatinine and (**A**) in-hospital mortality, (**B**) 90-day mortality, and (**C**) 1-year mortality. Estimates are presented as crude (green) and adjusted (black) hazard ratios, with 95% confidence intervals (CI)

In unadjusted Cox regression analysis, admission creatinine (log₂-transformed) was significantly associated with in-hospital mortality (HR 1.48, 95% CI: 1.30–1.69, *p* < 0.001). However, after adjustment for competing risk factors, this association was no longer statistically significant (adjusted HR 1.07, 95% CI: 0.92–1.26, *p* = 0.373) (Fig. 2A).

In contrast, admission creatinine consistently predicted mid- and long-term outcomes even after adjustment for potential confounders. For 90-day mortality, unadjusted Cox regression analysis showed a significant association with increased mortality (HR 1.52, 95% CI: 1.35–1.72, p < 0.001), which remained significant after adjustment (adjusted HR 1.16, 95% CI: 1.01–1.34, *p* = 0.039) (Fig. 2B). Similarly, higher admission creatinine was associated with increased 1-year mortality risk in both unadjusted and adjusted models (unadjusted HR 1.51, 95% CI: 1.36–1.67, *p* < 0.001; adjusted HR 1.20, 95% CI: 1.06–1.36, *p* = 0.004) (Fig. 2C). Admission creatinine remained significantly associated with 1-year mortality in a sensitivity analysis with additional adjustment for the incidence of acute kidney injury or RRT (Supplementary Table S8 and S9).

### Association of admission creatinine with length of hospital stay and short-term adverse kidney outcomes

**Fig. 3:**
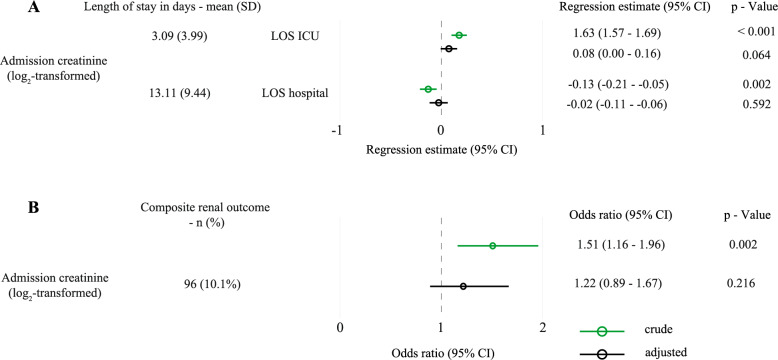
Association of admission creatinine with length of stay and short-term adverse kidney outcomes. Forest plots showing the association of admission creatinine with (**A**) hospital as well as ICU length of stay (LOS), and (**B**) the composite kidney outcome of de novo eGFR < 30 mL/min/1.73 m^2^ or requirement of kidney replacement therapy at discharge. Estimates are presented as crude (green) and adjusted (black) beta coefficients (**A**) or odds ratios (**B**), with 95% confidence intervals (CI)

Linear regression models adjusted for potential confounders demonstrated that higher admission creatinine values were not associated with prolonged ICU (adjusted difference 0.08 days, 95% CI: 0.00–0.16, *p* = 0.064) or hospital stays (−0.02 days, 95% CI: –0.11–0.06, *p* = 0.592) (Fig. 3A). Also, admission creatinine was only associated with the composite kidney outcome, defined as de novo eGFR < 30 mL/min/1.73m^2^ or need for dialysis at discharge, in the unadjusted analysis (OR 1.51, 95% CI: 1.16–1.96, *p* = 0.002) and there was no significant association after adjustment (adjusted OR 1.22, 95% CI: 0.89–1.67, *p* = 0.216, Fig. 3B).

### Admission creatinine and prediction scores for in-hospital mortality

**Fig. 4:**
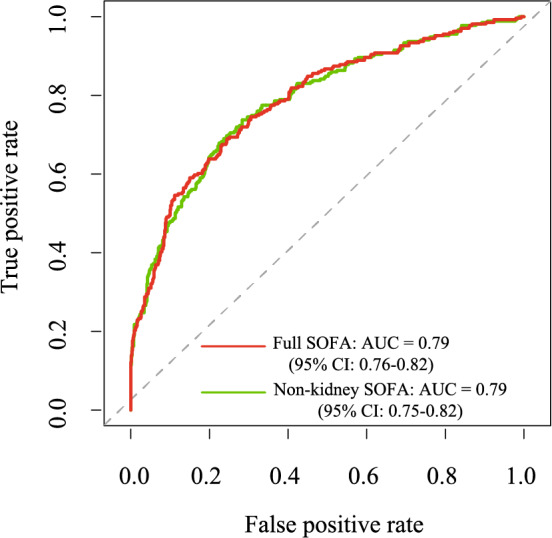
Performance of SOFA score for in-hospital mortality with and without inclusion of admission creatinine. Receiver operating characteristic curves showing the predictive performance for in-hospital mortality of the Full SOFA score including admission creatinine (red line) and a non-kidney SOFA score without admission creatinine (green line) including area under the curve (AUC) values with 95% confidence intervals (CI). SOFA: Sequential Organ Failure Assessment

To assess the use of admission creatinine in established prediction tools for in-hospital mortality, we compared the discriminative performance of the SOFA score with admission creatinine (full SOFA) and without it (non-kidney SOFA). The AUC was 0.79 (95% CI: 0.76–0.82) for the full SOFA and 0.79 (95% CI: 0.75–0.82) for the non-kidney SOFA score, indicating similar discrimination abilities of the two versions (Fig. 4).

### Predictive ability of pre-admission versus admission creatinine

**Fig. 5:**
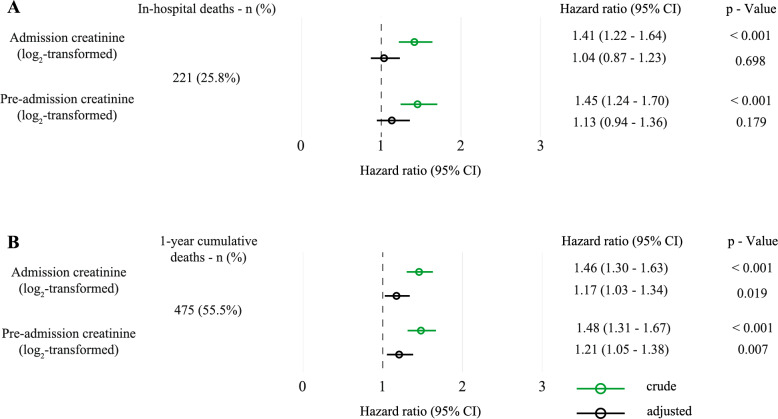
Comparison of pre-admission versus admission creatinine for the association with mortality. Forest plots showing the associations of admission creatinine and pre-admission creatinine with (**A**) in-hospital and (**B**) 1-year mortality. Estimates are presented as crude (green) and adjusted (black) hazard ratios with 95% confidence intervals (CI)

In a subgroup of patients with available pre-admission and admission creatinine values (*n* = 856), we examined whether prediction of mortality differed by pre-admission and admission creatinine (Fig. 5). Baseline characteristics and disease course for this subgroup are presented in Supplementary Tables S5 and S6. Both admission creatinine (adjusted HR 1.04, 95% CI: 0.87–1.23, *p* = 0.698) and pre-admission creatinine (adjusted HR 1.13, 95% CI: 0.94–1.36, *p* = 0.179) were not associated with in-hospital mortality after adjustment. In contrast, 1-year mortality was equally associated with both admission creatinine (adjusted HR 1.17, 95% CI: 1.03–1.34, *p* = 0.019) and pre-admission creatinine (adjusted HR 1.21, 95% CI: 1.05–1.38, *p* = 0.007). There was also no difference in the incidence rate and severity of AKI, when using the admission or the pre-admission creatinine as reference indicator of chronic kidney function (Supplementary Table S7). AKI was consistently associated with 1-year mortality, regardless of whether admission or pre-admission creatinine was defined as the baseline. The association with in-hospital mortality was slightly weaker when pre-admission creatinine was used as the baseline (Supplementary Table S7).

## Discussion

In this large, retrospective cohort study of ICU patients aged ≥ 90 years, we investigated the prognostic utility of admission serum creatinine in predicting short- and long-term outcomes. We found that admission creatinine was not independently associated with in-hospital mortality or other short-term endpoints but reliably predicted 1-year mortality. This lack of short-term prognostic value is further illustrated by the unchanged performance of the SOFA score when excluding its kidney component. In contrast, its association with mid- and long-term outcomes suggests a closer link of admission creatinine to underlying chronic kidney function than to acute illness severity. Finally, baseline kidney function determined by outpatient creatinine measurements was not superior to admission creatinine in predicting outcomes or define AKI.

Previous studies have reported that higher admission creatinine levels are independently associated with increased in-hospital mortality [13–15], but most have focused on mixed-age cohorts. Our data indicate that these associations may not hold in critically ill patients aged ≥ 90 years. This discrepancy may be explained by altered creatinine dynamics and selective ICU admission practices in very old adults. Several studies have shown that older adults experience a smaller rise in creatinine during AKI compared with younger individuals, mostly due to reduced muscle mass and diminished creatine turnover, leading to underestimation of acute kidney impairment [21]. While these factors also affect baseline creatinine levels, their impact becomes particularly pronounced during episodes of substantial change in kidney function, such as AKI [17, 22]. In addition to this attenuated peak response, older adults also show delayed creatinine kinetics, which further weakens its value as a real-time marker of acute illness severity [23]. Because of the altered creatinine dynamics, a high admission creatinine in very old patients more likely reflects chronic kidney dysfunction than acute illness severity. CKD, in turn, is known to be more predictive of long-term rather than short-term outcomes. Finally, ICU triage and treatment practices in the oldest may also contribute to these findings. Nonagenarians are often admitted to ICUs with less severe acute illness than younger individuals [24]. This pattern was also observed in our cohort, where the rate of ICU admissions after elective surgery was significantly higher as typically reported for younger adults [4, 24]. In such scenarios, admission creatinine values are also more likely to reflect a chronic state rather than an acute change due to critical illness [25, 26]. Together, these factors may explain why admission creatinine predicts long-term but not in-hospital mortality in very old patients. Thus, the limited short-term prognostic value of creatinine observed in our study suggests that future research should explore biomarkers presumably more reflective of acute kidney injury or less influenced by age-related physiological changes, such as cystatin C or emerging biomarkers of kidney stress and injury, including Dickkopf-3 (DKK3) [27].

Our analysis further demonstrates that omitting the kidney component from the SOFA score did not reduce its accuracy for in-hospital mortality prediction. This finding is consistent with our multivariable analyses showing no independent association between admission creatinine and in-hospital mortality. In nonagenarians, short-term outcomes may be more strongly driven by acute respiratory or hemodynamic failure, which are captured by other components of the SOFA score.

In contrast, admission creatinine remained independently associated with mid- and long-term mortality, suggesting that in very old ICU patients creatinine may primarily reflect chronic kidney dysfunction and overall physiological vulnerability rather than acute illness severity.

Moreover, the comparable prognostic performance of admission and pre-admission creatinine for both AKI and mortality underscore that obtaining historical creatinine values may not meaningfully enhance prediction in patients aged ≥ 90 years. These findings collectively support an age-adapted interpretation of kidney biomarkers and caution against uncritical application of standard prognostic tools in very old ICU populations. From a clinical perspective, these results suggest that in ≥ 90-year-old ICU patients, elevated creatinine at admission should not automatically be interpreted as a marker of poor short-term prognosis or limited benefit from ICU treatment. Instead, it may be more valuable for identifying individuals at increased long-term mortality risk. This distinction could guide discussions with patients and families, focusing on realistic expectations beyond hospital discharge and on aligning interventions with patient-centered goals.

Our study has several strengths, including the analysis of a large, well-characterized cohort of very old ICU patients, a population often underrepresented in critical care research. The availability of long-term follow-up data allowed assessment of short- and long-term mortality. Additionally, comparing admission and pre-admission creatinine in many patients provides clinically relevant insights for settings where pre-admission values are frequently unavailable. However, the retrospective, single-center design may also limit generalizability due to local ICU admission practices and the predominantly Caucasian population. Although we adjusted for multiple covariates, residual confounding cannot be excluded. In particular, frailty, sarcopenia and functional performances—which strongly influence outcomes in very old patients—were not directly assessed in this cohort. These aspects are only addressed by surrogates including age, comorbidity burden (CCI), BMI, and functional status at admission (GCS). At the same time, the analysis may be subject to overadjustment, despite careful covariate selection informed by literature and biological plausibility. Further, additional biomarkers of kidney function, such as cystatin C and urine albumin-creatinine ratio, were unavailable. Finally, while mortality outcomes were available up to one year, data on post-discharge quality of life and functional independence were lacking, although crucial for evaluating the benefits of intensive care in this age group [28].

## Conclusions

In conclusion, admission creatinine is a robust predictor for long-term mortality after ICU treatment but has limited value in predicting in-hospital outcomes in very old adults. Pre-admission creatinine does not improve prognostic accuracy or alter AKI classification in this population. Future studies investigating creatinine-based prediction scores for long-term outcomes in very old ICU patients are needed and might help to improve shared decision making.

## Supplementary Information

Supplementary Material 1.

## Data Availability

Data will be shared upon reasonable request to the corresponding authors.

## Abbreviations

AKI: Acute kidney injury
AUC: Area under the curve
BMI: Body mass index
CCI: Charlson Comorbidity Index
CI: Confidence interval
CPR: Cardiopulmonary resuscitation
eGFR: Estimated glomerular filtration rate
GCS: Glasgow Coma Scale
HR: Hazard ratio
ICU: Intensive care unit
KDIGO: Kidney disease: improving global outcomes
LOS: Length of stay
MAP: Mean arterial pressure
OR: Odds ratio
PDMS: Patient data management system
KRT: Kidney replacement therapy
SD: Standard deviation
SOFA: Sequential organ failure assessment
