# Supplementary material for: Admission creatinine and outcomes in very elderly critically ill patients: a retrospective cohort study
Source: BMC Geriatr. 2026 Jun 10;26:822. doi: 10.1186/s12877-026-07793-0 (PMC13255386; doi:10.1186/s12877-026-07793-0)
Supplement: Supplementary file 1 — Supplementary Material 1. [file 12877_2026_7793_MOESM1_ESM.pdf]

Supplement. Table of Contents

	Page
**Supplementary Figure S1.**<br>Patient disposition	2
**Supplementary Table S1.**<br>Number of missing values	3
**Supplementary Table S2.**<br>Baseline characteristics of included and excluded patients	4
**Supplementary Figure S2.**<br>Restricted cubic spline modeling of the association between admission creatinine and 1-year mortality	5
**Supplementary Table S3.**<br>Comparison of model fit between linear and restricted cubic spline modelling for admission creatinine	6
**Supplementary Table S4.**<br>SOFA Score with and without renal component on in-hospital mortality	7
**Supplementary Table S5.**<br>Baseline characteristics of the sub-cohort of individuals with available admission and pre-admission creatinine	8
**Supplementary Table S6.**<br>Procedures applied during ICU treatment in the sub-cohort of individuals with available admission and pre-admission creatinine	9
**Supplementary Table S7.**<br>AKI incidence and association with mortality for different definitions of baseline kidney function	10
**Supplementary Table S8.**<br>Sensitivity analysis with additional adjustment for renal replacement therapy	11
**Supplementary Table S9.**<br>Sensitivity analysis with additional adjustment for acute kidney injury	12

Supplementary Figure S1. Patient disposition

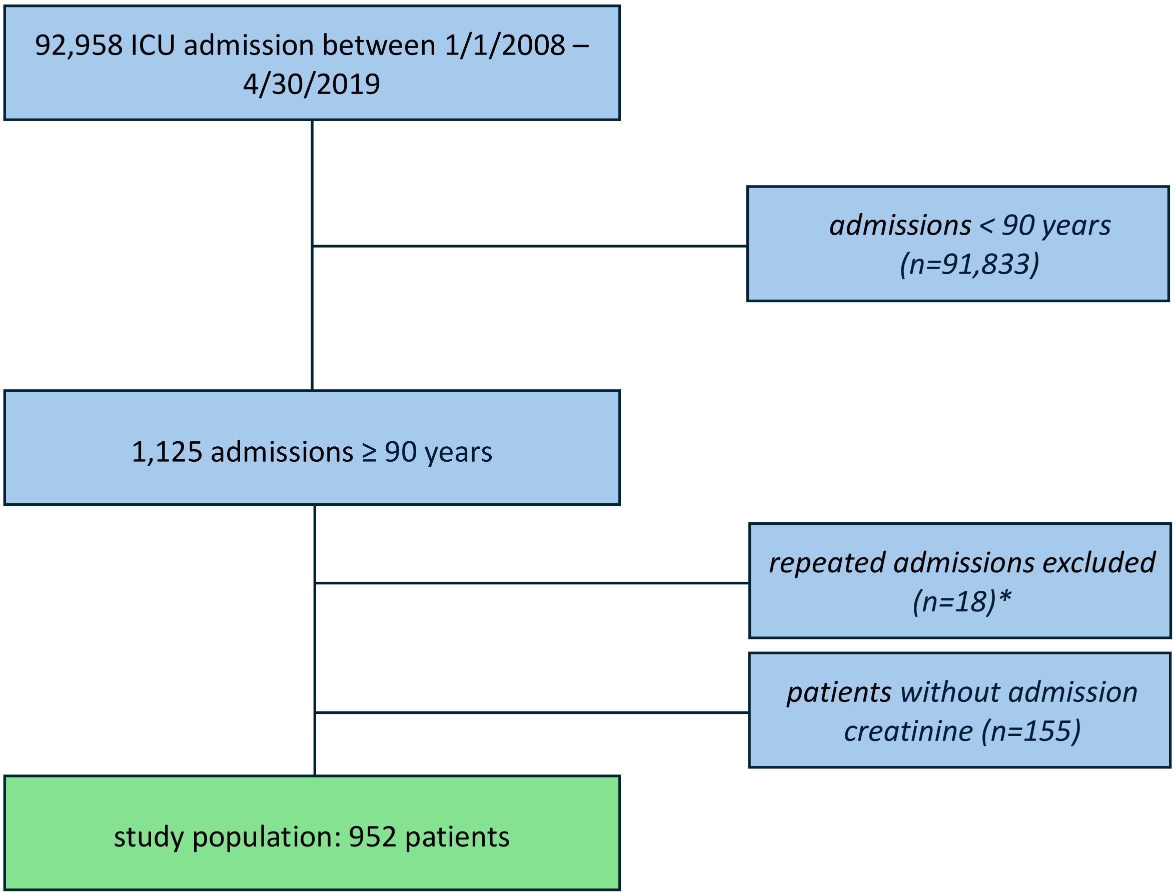

*Only the first ICU admission per patient was included.

Supplementary Table S1. Number of missing values

Characteristics – n (%)	Overall (n = 952)
Demographics	
Age	0
Female sex	0
BMI	279 (29.3)
Comorbidities	0
Primary admission	0
General presentation at admission	
GCS	0
Temperature	171 (17.9)
MAP	3 (0.3)
Heart rate	8 (0.8)
Laboratory results at admission	
Pre-admission creatinine	96 (10.1)
Admission creatinine	0 (0)
Hemoglobin	38 (4)
White blood cell count	27 (2.8)
Platelet count	12 (1.3)
Sodium	35 (3.7)
Bilirubin	237 (24.9)
pH	32 (3.4)
Lactate	31 (3.3)
Medication at admission	23 (2.4)
Disease course characteristics	0

BMI body mass index, *GCS* Glasgow Coma Scale, *MAP* mean arterial pressure

Supplementary Table S2. Baseline characteristics of included and excluded patients

Characteristics	Included (n = 952)	Excluded (n = 155)	p - Value
Demographics			
Age in years – median (IQR)	92.2 (3.1)	92.9 (2.8)	< 0.001
Female sex – n (%)	635 (66.7)	111 (71.6)	0.26
Comorbidities			
Charlson comorbidity index – median (IQR)	1 (2)	1 (1)	0.897
Hypertension – n (%)	674 (70.8)	100 (64.5)	0.137
Atrial fibrillation – n (%)	373 (39.2)	53 (34.2)	0.274
Cerebrovascular disease – n (%)	145 (15.2)	26 (16.8)	0.709
Diabetes – n (%)	129 (13.6)	21 (13.6)	0.966
Peripheral vascular disease – n (%)	84 (8.8)	17 (11.0)	0.478
COPD – n (%)	72 (7.6)	11 (7.1)	0.968
Primary admission			< 0.001
Medical – n (%)	303 (31.8)	75 (48.7)	
Elective surgery – n (%)	360 (37.8)	48 (31.2)	
Emergency surgery – n (%)	289 (30.4)	31 (20.1)	
General presentation at admission			
GCS – median (IQR)	15.0 (3)	15.0 (2)	0.001
MAP in mmHg – median (IQR)	85.0 (30.2)	90 (29.0)	0.066
Heart rate in bpm – median (IQR)	80 (37)	79 (34)	0.925
Laboratory results at admission			
Sodium in mmol/L – median (IQR)	140 (5)	139 (5)	0.613
Lactate in mmol/L– median (IQR)	1.1 (0.9)	1.3 (2.1)	< 0.001

IQR interquartile range, *COPD* chronic obstructive pulmonary disease, *GCS* Glasgow Coma Scale, *MAP* mean arterial pressure, *bpm* beats per minute

Baseline characteristics are presented for patients included in the study and those excluded due to missing admission creatinine values.

Supplementary Figure S2. Restricted cubic spline modeling of the association between admission creatinine and 1-year mortality

A.

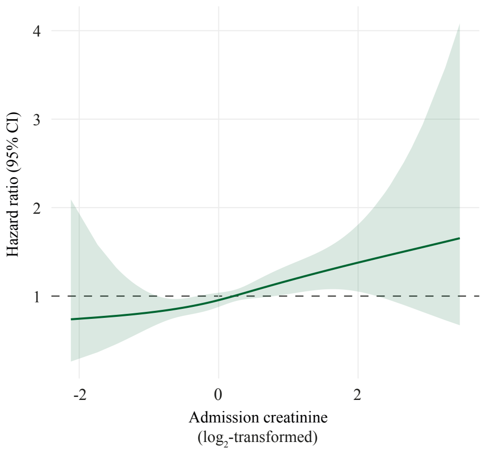

B.

Creatinine spline terms	Coefficient (β)	p - Value
1	0.50	0.09
2	0.79	0.49
3	0.75	0.17

(A) Adjusted hazard ratio (HR) for 1-year mortality by admission creatinine (log_2-transformed) based on a Cox proportional hazards model with restricted cubic splines (3 degrees of freedom). The solid line represents the estimated HR relative to the median creatinine (HR = 1, dashed line), and the shaded area the 95 % confidence interval. (B) Corresponding spline term coefficients (β) and p-values from the model.

Supplementary Table S3. Comparison of model fit between linear and restricted cubic spline modelling for admission creatinine

Model	[1] $\Delta\chi^2$	[2] Δdf	p - Value
Non-Linear vs Linear	0.09	2	0.95

[1] $\Delta\chi^2$ = chi-square statistic for the likelihood-ratio test comparing the spline and linear model
[2] Δdf = difference in the number of degrees of freedom between the two models

Cox proportional hazards models for 1-year mortality were fitted separately and compared, with admission creatinine modeled either as a nonlinear variable using restricted cubic splines or as a linear variable.

Supplementary Table S4. Association of the SOFA score with and without the renal component and in-hospital mortality

Variables	Odds ratio	95% CI	p - Value
full SOFA			
crude	2.09	1.82 – 2.42	< 0.001
adjusted	1.91	1.64 – 2.24	< 0.001
Non-kidney SOFA			
crude	1.84	1.63 – 2.10	< 0.001
adjusted	1.79	1.56 – 2.06	< 0.001

CI confidence interval, *SOFA* sequential organ failure assessment

Odds ratios (ORs) with 95% confidence intervals (CIs) are derived from logistic regression models assessing the association between the Sequential Organ Failure Assessment (SOFA) score and in-hospital mortality. Crude models are unadjusted, whereas adjusted models include established confounders: age, sex, body mass index (BMI), Charlson Comorbidity Index (CCI), admission type, Glasgow Coma Scale (GCS), lactate, bilirubin, mean arterial pressure (MAP), use of vasopressors, and mechanical ventilation at ICU admission. The non-kidney SOFA score excludes the renal (creatinine) component.

Supplementary Table S5. Baseline characteristics of sub-cohort of individuals with available admission and pre-admission creatinine

Characteristics	Overall (n = 856)
Demographics	
Age in years – median (IQR)	92.2 (3.2)
Female sex - n (%)	577 (67.4)
BMI in kg/m^2 – median (IQR)	23.4 (3.9)
Comorbidities	
Charlson comorbidity index – median (IQR)	1 (2)
Hypertension – n (%)	601 (70.2)
eGFR <60 mL/min/1.73 m² – n (%)	581 (67.9)
Atrial fibrillation - n (%)	330 (38.6)
Cerebrovascular disease – n (%)	130 (15.2)
Diabetes- n (%)	129 (13.6)
Peripheral vascular disease - n (%)	75 (8.8)
COPD - n (%)	64 (7.5)
Primary admission	
Medical – n (%)	259 (30.3)
Elective surgery – n (%)	331 (38.7)
Emergency surgery – n (%)	266 (31.1)
General presentation at admission	
GCS – median (IQR)	15.0 (3)
Temperature in degrees Celsius – median (IQR)	36.2 (1)
MAP in mmHg – median (IQR)	85.0 (30)
Heart rate in bpm – median (IQR)	79.0 (32)
Laboratory results at admission	
Hemoglobin in g/dL – median (IQR)	10.3 (2.2)
White blood cell count in 10^3/microliter – median (IQR)	10.7 (6.3)
Platelet count in 10^3/microliter – median (IQR)	210 (114)
Sodium in mmol/L – median (IQR)	140 (5)
Creatinine in mg/dL – median (IQR)	1.1 (0.8)
Bilirubin in mg/dL – median (IQR)	0.7 (0.6)
pH – median (IQR)	7.38 (0.09)
Lactate in mmol/L – median (IQR)	1.1 (0.9)

IQR interquartile range, *BMI* body mass index, *COPD* chronic obstructive pulmonary disease, *GCS* Glasgow Coma Scale, *MAP* mean arterial pressure, *bpm* beats per minute

Supplementary Table S6. Procedures applied during ICU treatment in the sub-cohort of individuals with available admission and pre-admission creatinine

Characteristics	Overall (n = 856)
Procedures	
Mechanical ventilation - n (%)	293 (34.2)
Vasopressor therapy - n (%)	371 (43.3)
Surgery during ICU stay - n (%)	7 (0.8)
CPR – n (%)	30 (3.5)
KRT – n (%)	29 (3.4)

ICU intensive care unit, *CPR* cardiopulmonary resuscitation, *KRT* kidney replacement therapy

Supplementary Table S7. AKI incidence and association with mortality for different definitions of baseline kidney function

	Pre-admission Creatinine			Admission Creatinine		
	n (%)	*Association to in-hospital mortality*[a]	*Association to 1-year mortality*[a]	n (%)	*Association to in-hospital mortality*[a]	*Association to 1-year mortality*[a]
**AKI – Total (n)**	213 (100)	1.24 (0.93-1.64, 0.148)	1.46 (1.18-1.80, < 0.001)	188 (100)	1.37 (1.03-1.82, 0.030)	1.35 (1.10-1.66, < 0.004)
**AKI Stage 1 (%)**	144 (67.6)			128 (68.1)		
**AKI Stage 2 (%)**	31 (14.6)			24 (12.8)		
**AKI Stage 3 (%)**	38 (17.8)			36 (19.1)		

[a] presented as hazard ratios with 95% CIs
AKI acute kidney injury, *CI* confidence interval

Incidence and severity of acute kidney injury (AKI) and its association with mortality according to different definitions of baseline kidney function (pre-admission vs. admission creatinine). AKI was defined according to KDIGO criteria. Associations with in-hospital mortality and with 1-year mortality are presented as hazard ratios (HRs) with 95% confidence intervals (CIs). Regression models were adjusted for established confounders including: age, sex, body mass index (BMI), Charlson Comorbidity Index (CCI), admission type, Glasgow Coma Scale (GCS), lactate, bilirubin, mean arterial pressure (MAP), use of vasopressors, and mechanical ventilation at ICU admission..

Supplementary Table S8. Sensitivity analysis with additional adjustment for renal replacement therapy

Outcomes	Hazard ratio	95% CI	p - Value
In-hospital mortality	1.05	0.89 – 1.24	0.548
90-day mortality	1.12	0.97 – 1.30	0.127
1-year mortality	1.17	1.03 – 1.33	0.019

CI confidence interval

Sensitivity analysis evaluating the association between admission creatinine and mortality outcomes with additional adjustment for renal replacement therapy (RRT). Hazard ratios (HRs) with 95% confidence intervals (CIs) are derived from Cox proportional hazards regression models adjusted for established confounders as described in the main analysis.

Supplementary Table S9. Sensitivity analysis with additional adjustment for acute kidney injury

	**Pre-admission Creatinine**			**Admission Creatinine**		
	Hazard ratio	*95% CI*	*p – Value*	Hazard ratio	*95% CI*	*p – Value*
In-hospital mortality	1.01	0.85-1.20	0.936	1.12	0.93-1.34	0.219
90-day mortality	1.13	0.97-1.31	0.128	1.16	0.99-1.35	0.065
1-year mortality	1.15	1.01-1.31	0.038	1.19	1.05-1.37	0.009

CI confidence interval

Sensitivity analysis evaluating the association between creatinine (pre-admission and admission) and mortality outcomes with additional adjustment for acute kidney injury (AKI). Hazard ratios (HRs) with 95% confidence intervals (CIs) are derived from Cox proportional hazards regression models adjusted for established confounders as described in the main analysis.
